# Health-seeking behaviour of older women: a protocol for a global scoping review

**DOI:** 10.1136/bmjopen-2026-127153

**Published:** 2026-11-02

**Authors:** Rakhi Ghoshal, Foram Gandhi, Ashutosh Garg, Jasleen Kaur, Vijna Boodhoo, Anita Gadgil, Nobhojit Roy

**Affiliations:** 1 WHO Collaborating Centre for Research in Surgical Care in LMICs, Kolkata, India; 2 Department of Obstetrics, Gynecology and Reproductive Sciences, Yale School of Medicine, New Haven, Connecticut, USA; 3 Department of General Medicine, Dr BR Ambedkar State Institute of Medical Sciences, Sahibzada Ajit Singh Nagar, Punjab, India; 4 Government Doon Medical College, Dehradun, Uttarakhand, India; 5 University of Glasgow, Glasgow, UK; 6 Program for Global Surgery and Trauma, The George Institute for Global Health India, New Delhi, India; 7 Cachar Cancer Hospital and Research Centre, Silchar, Assam, India; 8 Department of Global Public Health, Karolinska Institutet, Stockholm, Sweden; 9 The George Institute for Global Health India, New Delhi, India

**Keywords:** Aged, Health Services Accessibility, Patient Participation, Vulnerable Populations, Decision Making

## Abstract

**Abstract:**

**Background:**

Globally people are living longer and the proportion of older people with health problems is increasing. While women generally outlive men, their additional years are often marked by high levels of morbidity, even as their health needs remain comparatively under-recognised in research, policy and healthcare delivery. To compound this, health-seeking behaviour of older women is also shaped by multiple interacting individual, household, community, socioeconomic and health-system factors that work to delay or hinder timely access to healthcare. The scoping review aims to identify and map factors influencing health-seeking behaviour among women aged 60 years and older globally, examine how these factors are conceptualised across different settings and identify gaps in the existing evidence. This article is the protocol for the proposed scoping review.

**Methods and analysis:**

Studies published in English from 2021 onwards will be identified through searches of PubMed, Scopus, PsycINFO, IBSS, AgeLine and Google Scholar. Eligible studies will include women aged 60 years and more, from all geographical settings and for all health conditions and will identify all factors influencing health-seeking behaviour. Data will be extracted using a standardised extraction framework. Findings will be synthesised using two complementary frameworks: Andersen’s Behavioural Model of Health Services Use, which categorises predisposing, enabling and need factors, and Penchansky and Thomas’s Five Dimensions of Access, which is adapted to include ‘awareness’ as a sixth dimension. The review will be reported using the PRISMA-ScR reporting guidelines.

**Ethics and dissemination:**

This scoping review will synthesise data from publicly available published studies and will not involve primary data collection from human participants or access to identifiable individual-level data; thus, ethical approval is not required. The findings will be disseminated through a peer-reviewed publication and may also be shared through academic and policy forums relevant to ageing, women’s health and healthcare access.

**Strengths and limitations of this study**

- This is a comprehensive review of databases—including AgeLine that is dedicated to evidence on older populations.
- This review integrates Andersen’s model with an adapted Penchansky and Thomas Five Dimensions of Access framework (adds the sixth A) that helps distinguish individual and social determinants from health-system access factors, instead of an unstructured list of barriers to health-seeking.
- The data extraction sheet will be pilot tested across three pairs of reviewers, where each pair shall extract data from a few studies; the extracted data shall be refined to ensure consistency. The entire data extraction process shall be followed through with periodic audits. At the end, a select random sample of extracted studies will be reviewed by a second co-author to assess the accuracy and consistency of the extracted data. In the absence of formal quality assessment, this shall ensure rigour.
- The 5-year window excludes earlier evidence, and publications in other languages are not part of this review.

## Background

 Old age provides a complex convergence of health issues: age-related chronic conditions, increased risk of accidents such as falls and acute health problems. People, globally, are living longer, implying that the proportion of older people facing regular to aggravated health problems is correspondingly increasing.^1^ Right before the pandemic hit us, the number of people aged 60 years and more outnumbered children under the age of 5, and this trend is expected to continue, and by 2030, one in six people shall be over the age of 60 years.^2^

Of these growing numbers of older people, women constitute a majority—as in most countries they outlive men. The World Bank estimates that in low-income countries, women shall live 7 years more than their mothers did.^3^ More than half the global older population is female^4^—a demographic trait that is referred to as the feminisation of ageing.^5^ However, despite this specifically feminised older population, the health needs of older women are significantly under-prioritised and under-represented.^5^

Conditions like osteoporosis, cardiovascular diseases, dementia and depression figure disproportionately among older women.^6–9^ Residual effects of physiological changes during women’s reproductive life can add complications to it. However, these unique healthcare needs have not found their due place in policy, research or healthcare delivery. For several government programmes, the focus on women peaks when it comes to reproductive and maternal health but fades for women who are older. This is what creates the morbidity-mortality paradox^10^—a state where women’s mortality is reduced, and they live longer, but since they lack appropriate healthcare, they end up having to live with increased morbidities through their extra years.

The odds of not receiving timely healthcare increase for women in poorer households, in families without insurance and for women in rural areas where availability of regular healthcare is irregular.^11 12^ Further to these structural intersecting factors, women themselves—especially in communities with stricter gender norms, that normalise women’s pain and place reduced value on the lived experiences of those older—fail to seek and access timely healthcare. Older women are hesitant to actively seek healthcare or express the need for it, owing to constrained financial reserves or their own lack of decision-making capability,^13–15^ leading to a growing volume of unmet health needs. Barriers affect the need, intention, decision and capacity to seek timely and relevant healthcare for and by older women and contribute to the morbidity-mortality paradox.

### Study rationale

Health-seeking behaviour is a dynamic process shaped by the interaction between individuals, their households and social environments, and the health system. Levesque *et al*^16^ conceptualise healthcare access in terms of people’s abilities to perceive, seek, reach, purchase and engage with services, and the corresponding characteristics of health systems. Health-seeking involves recognising a health need, deciding whether and when to seek care, identifying appropriate services and mobilising the resources and support required to access them. The availability and accessibility of services, healthcare infrastructure and provider attitudes and practices can further influence if and when, people seek healthcare and return for follow-up. Understanding health-seeking behaviour therefore requires understanding the individual, social, economic and structural factors that interact and shape decision-making and access.

These interactions are particularly important for older women, whose health-seeking behaviour is likely shaped by the intersection of gender and age, alongside socioeconomic circumstances, household relationships and the availability and responsiveness of healthcare services. Family support and caregiving responsibilities, financial dependence, cultural norms surrounding women’s health needs, awareness of available services, mobility and transportation constraints, and past experiences with healthcare providers all influence whether and when, older women seek care.^17–19^ Examining health-seeking as more than an individual decision or a measure of healthcare utilisation is therefore essential to understanding the barriers older women face in recognising their needs, deciding to seek care and accessing appropriate services.

A systematic review^20^ of the social determinants of healthcare access among rural older women aged 60 years and above, synthesised 38 studies to identify individual, healthcare, socioeconomic and sociocultural influences shaping health-seeking behaviour. However, the review only included quantitative studies in which older women constituted at least 10% of the study population and their healthcare determinants were reported separately; also, the scope was restricted to rural settings. The review did not map the broader range of evidence that may be available on older women’s health-seeking across all settings, which could illuminate how women experience, interpret and navigate barriers to care across contexts. Since this review in 2017, population ageing, healthcare delivery and requirement of care and access, including those associated with the COVID-19 pandemic,^21^ are expected to have sufficiently altered to now merit another exploration into this critical topic.

### Study objectives

This scoping review will study the factors that globally inform health-seeking behaviour of women aged 60 years and more. It will examine and list the factors operating at individual, interpersonal, household, community, health-system and broader structural levels to identify stage-wise barriers that impinge health-seeking, across the spectrum; viz. from recognising a health need, through decision-making around seeking care and being able to access care. By identifying the scope and characteristics of existing evidence around this question, this review will help clarify current knowledge, highlight under-researched populations and contexts, and establish priorities for future research, policy and interventions to improve equitable healthcare access for older women.

This article is the protocol for the proposed scoping review.

### Conceptual model

This review will be guided by two complementary conceptual frameworks: Andersen’s Behavioural Model of Health Services Use and Penchansky and Thomas’s Five Dimensions of Access (5As) framework.

Andersen’s Behavioural Model conceptualises health service utilisation as the function of three broad determinant categories, viz. predisposing factors, enabling factors and need factors.^22^ Predisposing factors include demographic characteristics, education, beliefs, social norms and other attributes that influence an individual’s likelihood and ability to seek healthcare. Enabling factors refer to the resources that facilitate or constrain access to health services, such as household income, health insurance, transportation, family support and availability of health facilities. Finally, need factors encompass perceived needs whereby individuals recognise symptoms or ill health, and evaluated need as assessed by health professionals. Together, these categories recognise that healthcare utilisation is not determined by health status alone but by the interaction between personal, social and structural circumstances.

However, while Andersen’s model explains why individuals seek—or do not seek—healthcare, it does not explain how characteristics of health services themselves influence access. To understand this component, the review will draw on Penchansky and Thomas’s 5As framework. This framework conceptualises healthcare access as the ‘degree of fit’ between healthcare services and the population they serve.^23^ This framework comprises five dimensions: (a) availability (adequacy of healthcare resources), (b) accessibility (geographical location and transportation), (c) affordability (financial costs relative to ability to pay), (d) organisation and delivery of services, and (e) acceptability (the compatibility of healthcare services with patients’ cultural values, preferences, gender norms and expectations). Saurman^24^ added the sixth component to this framework, which is ‘Awareness’ about available services. Awareness is a function of effective communication and information strategies including health literacy, which prevents or facilitates people from seeking timely and appropriate healthcare. In the absence of awareness, an older woman shall not be able to make the required health-seeking decision.

These two frameworks are complementary: Andersen’s model captures the broader social and behavioural determinants that shape older women’s decision-making process, whereas the adapted Penchansky and Thomas’s framework provides the model to understand the health-system characteristics that either facilitate or impede access. When combined, the frameworks allow us to distinguish between barriers arising from women’s individual circumstances, family and community environments, and those originating within the health systems and influencing demand-side health-seeking behaviour. The combined model will help identify and distinguish factors that are potentially modifiable through health policy and service delivery, versus those that would require broader social or economic interventions; they would also help understand where and when multiple determinants interact to influence health-seeking behaviour of older women across the globe.

## Methods and analysis

### Search strategy and information sources

The team is developing a robust and reproducible search strategy in accordance with established scoping review methodology—guided by the Joanna Briggs Institute (JBI) for scoping reviews. The Population–Concept–Context framework will help combine relevant keywords and database-specific free-text terms relating to older women and health-seeking behaviour. Once developed, it will be refined through preliminary searches and adapted for each of the selected databases. The team will document the complete search strategies, databases and interfaces searched, search dates and limits applied. The search process and review findings will be reported in accordance with relevant scoping review guidance, including Preferred Reporting Items for Systematic Reviews and Meta-Analyses extension for Scoping Reviews (PRISMA-ScR). Finalising the search strategy will be a three-stepped process:

1. An initial strategy to answer the research question will be consultatively and iteratively developed by all co-authors along with inputs from a senior researcher.
2. The strategy will be used to run a pilot search of the first 200 titles and abstracts, and based on a quick review of the yield, the search strategy shall be modified.
3. This version of the strategy shall be reviewed by an external subject matter expert; based on their feedback, the strategy will be finalised for the review.

The search strategy will be further adapted for the following databases: PubMed (PubMed), Scopus (Scopus), PsycINFO (Ovid), International Bibliography of the Social Sciences (ProQuest), AgeLine (EBSCOHost) and Google Scholar (using Publish or Perish). Refer to online supplemental file 1 for the rationale for these databases. Once all titles have been retrieved, they will be imported to Covidence, which is a web-based platform to organise search results, screening and data extraction for scoping and other reviews. Each abstract, as well as each full text, will be simultaneously screened by two co-authors; conflicts between decisions, in both stages, shall be resolved by a dedicated third reviewer. Refer to online supplemental file 1 for the complete search strategies for all databases, including filters and limits used.

### Study selection

#### Inclusion and exclusion criteria

The review will capture the global picture of health-seeking by older women in the last 5 years. Studies only in English will be included since the team lacks the resources required to reliably identify and/or translate publications in any other global languages. The team has developed the following inclusion and exclusion criteria to inform the search. Studies will be included if they fulfil the following criteria:

- P (Population): Women aged 60 years and above.
- I (Phenomenon of Interest (ie, health-seeking behaviour): Isolation, unemployment and cultural stigma, alongside enabling resources including physical accessibility, affordability and family escort support. We will also study acceptability of care as well as perceived needs stemming from symptom severity, functional limitations and chronic multimorbidity. All of these will be informed by Andersen’s and (updated) Penchansky and Thomas’ models.
- C (Context): any geography, any health condition that requires clinical attention or care.
- O (Outcomes): any measurable or reported outcome related to health-seeking behaviour, including barriers and enablers, utilisation rates, care-seeking pathways, delay intervals and qualitative accounts of care-seeking experiences and decision-making.

Studies will be excluded if they:

- Population: Focus exclusively on men (even if older), focus on healthcare providers’ attitudes, knowledge or clinical behaviour with no patient-level care-seeking data, or examine older women exclusively in their role as caregivers or in institutional settings (such as prisons).
- Phenomenon of interest: If no component of health-seeking behaviour is examined; purely pharmacological or any other trial, clinical treatment outcome studies, disease burden or epidemiological care-seeking' to 'heatlh-seeking.
- Study design: Case reports and case series; editorials, letters, opinion pieces or commentaries with no primary data reported; conference abstracts without full-text results; study protocols with no results reported; animal studies.
- Other: Preprints; published before 2021; non-English language publication with no English abstract; duplicate publication superseded by a full paper (in which case we shall retain the most complete version); studies with no extractable data for women aged 60 years and more; study protocols with no results reported or conference abstracts; studies focused solely on emergency or disaster settings; studies focused solely on healthcare providers’ attitudes and perceptions; studies focused solely on utilisation of healthcare and services.

The process of selecting studies is presented in figure 1.

**Figure 1:**
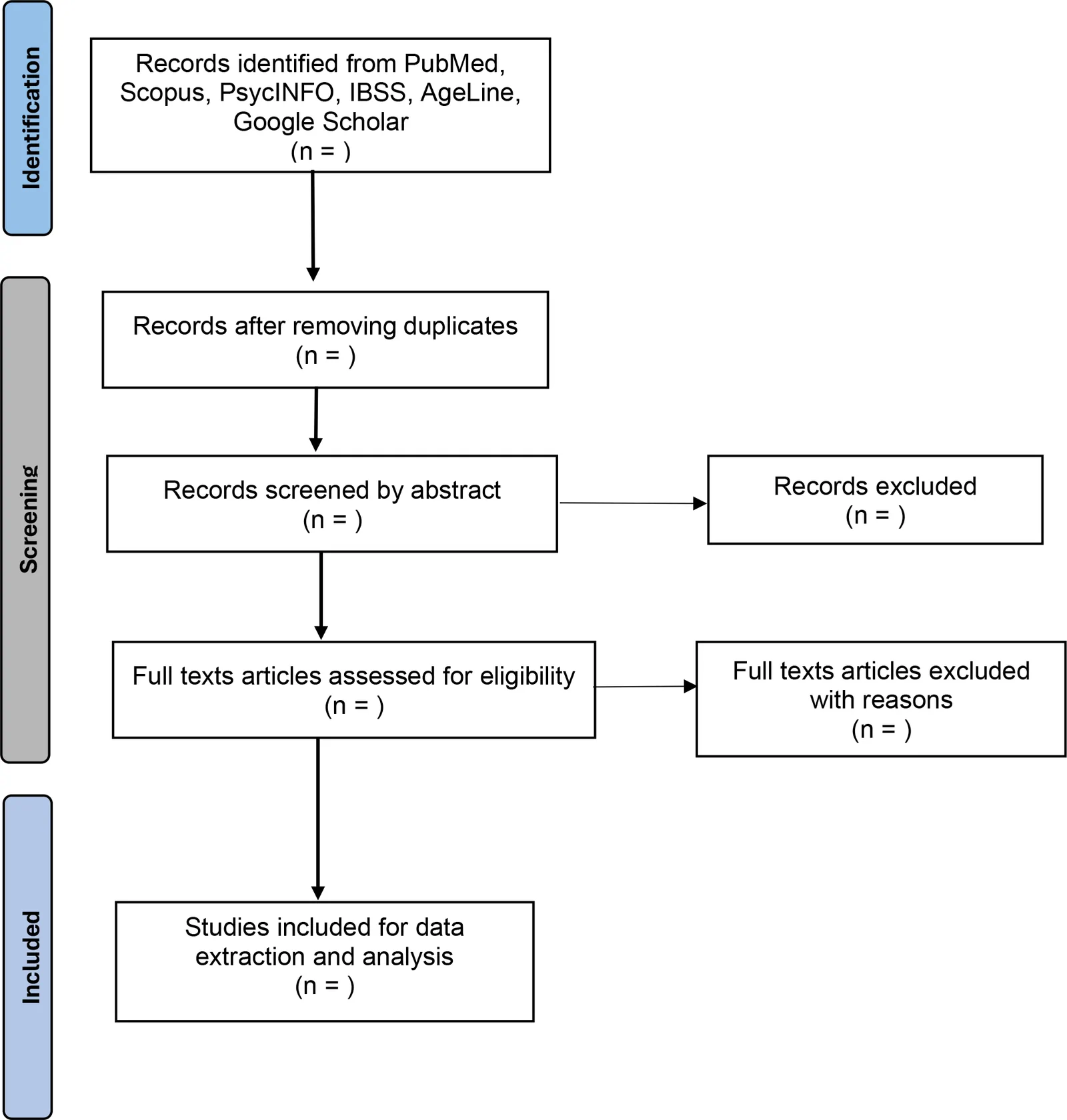
Preferred Reporting Items for Systematic Reviews and Meta-Analyses flow diagram describing the process of article review, inclusion and exclusion for the scoping review.

### Data extraction and management

A data extraction sheet, based on the synthesised conceptual frameworks, will be developed. Data extraction from each selected full-text article will be conducted in three stages. First, three teams of two co-authors will independently extract data from a small sample of studies to pilot the data extraction sheet. The teams will then convene to discuss discrepancies, assess consistency in the interpretation and application of the extraction criteria and revise the extraction items as necessary. Second, each selected study will be assigned to a single co-author for data extraction. Periodic internal audits will be conducted during this stage to monitor consistency and identify issues requiring clarification. Third, once data extraction is complete, a sample of extracted studies will be reviewed by a second co-author to assess the accuracy and consistency of the extracted data. Any disagreements that cannot be resolved through discussion between the reviewers will be referred to the designated third reviewer for resolution.

### Synthesising results

The extracted data shall be reviewed by the team to identify the different categories and levels of factors that influence health-seeking of older women. The review would also be able to indicate the factors that are predominant in high-resource settings versus those in low-resource ones; the review shall also identify the number and proportion of studies reporting specific characteristics, factors, outcomes and themes for older women and their health-seeking behaviour. The findings shall be contextualised with relevant literature. While reporting the results as a formal academic publication, the review will highlight evidence gaps and identify implications for policy, practice and research.

### Study status

As of 12 October 2026, the team has screened abstracts and is currently screening full texts. The study was conceptualised in April 2026 and is estimated to be completed by 31 March 2027.

### Ethics and dissemination

This scoping review will synthesise data only from publicly available published studies and will neither involve any form of primary data collection from human participants nor require any access to identifiable individual-level data. Thus, ethics approval is not required for this review. The protocol for this scoping review is registered with Open Science Framework (OSF) and is currently under embargo till 31 May 2027. The findings of the scoping review will be disseminated through a peer-reviewed publication and may also be shared through academic and policy forums relevant to ageing, women’s health and healthcare access.

## Discussion

The ongoing decade has been declared by the UN General Assembly as the UN Decade of Healthy Ageing, and the WHO has prioritised implementing strategies to improve the lives of older people. In the near future, older women shall outnumber any other demographic group globally—even as countries have failed to put the health spotlight on them. Against this backdrop, this review is expected to contribute in three important ways: first, it will synthesise evidence to identify areas where research is concentrated or lacking. Second, it will offer a structured classification of determinants influencing healthcare-seeking behaviour, facilitating comparison across different contexts and health systems. Finally, it will identify knowledge gaps that can guide future research and inform the design of policies and interventions aimed at improving healthcare access for older women.

## Supplementary material

10.1136/bmjopen-2026-127153 online supplemental file 1
