# Supplementary material for: Health-seeking behaviour of older women: a protocol for a global scoping review
Source: BMJ Open. 2026 Nov 2;16(11):e127153. doi: 10.1136/bmjopen-2026-127153 (PMC13735260; doi:10.1136/bmjopen-2026-127153)
Supplement: Supplementary file 1 [file bmjopen-2026-127153.reviewer_comments.pdf]

PEER REVIEW HISTORY

BMJ Open publishes all reviews undertaken for accepted manuscripts. Reviewers are asked to complete a checklist review form and are provided with free text boxes to elaborate on their assessment. These free text comments are reproduced below.

ARTICLE DETAILS

Title (Provisional)

Health-seeking behaviour of older women: a protocol for a global scoping review

Authors

Ghoshal, Rakhi; Gandhi, Foram; Garg, Ashutosh; Kaur, Jasleen; Boodhoo, Vijna; Gadgil, Anita; Roy, Nobhojit

---

VERSION 1 - REVIEW

**Reviewer**	**1**
**Name**	**Kabonga, Itai**
**Affiliation**	**CeSHHAR Zimbabwe**
**Date**	**23-Sep-2026**
**COI**	**none**

Health-seeking behaviour of older women: a protocol for a global scoping reviewbmjopen-2026-127153

Abstract

• The authors should consider renaming the “Introduction” section to “Background.”

• The reporting guidelines currently included in the Ethics and Dissemination section should be removed, as these belong in the Methods section. The same issue should be addressed on page 9.

Strengths and Limitations

• The authors identify the comprehensive database search as a strength, including AgeLine, which is specifically focused on evidence relating to older populations. This strength could be further enhanced by searching additional sources of grey literature, including relevant grey literature databases and search engines such as medRxiv, where appropriate.

• Limitation: The authors state that not conducting a quality assessment of the included studies is a limitation because it prevents grading the strength of the evidence. However, quality appraisal is not a standard requirement for scoping reviews. I therefore suggest removing this as a limitation.

Study Rationale

• I do not think the first paragraph of the Study Rationale adequately addresses the rationale for conducting the study. It should be revised to clearly explain why this review is needed, including the evidence gap that the review intends to address.

• The review questions should not be included within the Study Rationale. They should either be presented as a standalone section or moved to an appropriate section elsewhere in the manuscript.

Methods and Analysis

• The authors state: "The team shall develop a search strategy according to established scoping review methodology." This should be expanded by explicitly stating which scoping review methodology or framework will be followed. The authors should also clarify whether the search strategy will combine subject headings (e.g., MeSH terms) and free-text search terms, and explain how these will be structured.

• One way to assess the comprehensiveness of the search strategy is to identify indicator or sentinel publications that are known to be relevant to the review and test whether the proposed search strategy retrieves them.

• I strongly recommend involving a librarian/information specialist in developing and refining the search strategy.

• The authors should explain how conflicts or disagreements during the pilot data extraction process will be identified and resolved. For example, specify whether disagreements will be resolved through discussion, consultation with a third reviewer, or another predefined approach.

• The reporting of results is currently predominantly qualitative. The authors should consider incorporating more quantitative descriptive information, such as the number and proportion of studies reporting specific characteristics, factors, outcomes, or themes. This would provide a clearer numerical summary of the evidence and complement the qualitative synthesis.

VERSION 1 - AUTHOR RESPONSE

**Reviewer: 1**			
**Abstract**			
1	The authors should consider renaming the "Introduction" section to "Background."	Done	Introduction is now retitled 'Background' (in both, Abstract and the main ms.)

2	The reporting guidelines currently included in the Ethics and Dissemination section should be removed, as these belong in the Methods section. The same issue should be addressed on page 9.	Done	We have now removed reporting guidelines from Ethics and Dissemination section and placed them under Methods section (for both, abstract and main ms.)
**Strengths and Limitations**			
3	The authors identify the comprehensive database search as a strength, including AgeLine, which is specifically focused on evidence relating to older populations. This strength could be further enhanced by searching additional sources of grey literature, including relevant grey literature databases and search engines such as medRxiv, where appropriate.	We shall consider	Thank you for this suggestion; we shall try to search additional grey literature.
4	Limitation: The authors state that not conducting a quality assessment of the included studies is a limitation because it prevents grading the strength of the evidence. However, quality appraisal is not a standard requirement for scoping reviews. I therefore suggest removing this as a limitation.	Done	We have removed this point from the Limitations.
**Study Rationale**			
5	I do not think the first paragraph of the Study Rationale adequately addresses the rationale for conducting the study. It should be revised to clearly explain why this review is needed, including the evidence gap that the review intends to address.	Done	The rationale section has been updated in response to this comment.

6	The review questions should not be included within the Study Rationale. They should either be presented as a standalone section or moved to an appropriate section elsewhere in the manuscript.	Done	The review question is now under new sub-section titled – Study Objectives.
**Methods and Analysis**			
7	The authors state: "The team shall develop a search strategy according to established scoping review methodology."<br><br>This should be expanded by explicitly stating which scoping review methodology or framework will be followed.<br><br>The authors should also clarify whether the search strategy will combine subject headings (e.g., MeSH terms) and free-text search terms, and explain how these will be structured.	Done	The start of the Search Strategy section is now redrafted to elucidate the strategy that shall be used; a supplementary file is added which contains the strategy.
8	I strongly recommend involving a librarian/information specialist in developing and refining the search strategy.	We shall try to	We shall try to identify and involve a librarian as suggested.
9	The authors should explain how conflicts or disagreements during the pilot data extraction process will be identified and resolved. For example, specify whether disagreements will be resolved through discussion, consultation with a third reviewer, or another predefined approach.	Done	We have mentioned under Data extraction that 'In cases of disagreements in decision, a dedicated third reviewer shall resolve it, for both, title-abstract screening and full-text screening stages.'
10	The reporting of results is currently predominantly qualitative. The authors should consider incorporating more quantitative descriptive information,	Done	Thank you for this suggestion. We shall include this section; we have now mentioned this

	such as the number and proportion of studies reporting specific characteristics, factors, outcomes, or themes. This would provide a clearer numerical summary of the evidence and complement the qualitative synthesis.		in the Synthesizing results sub section.
