# Supplementary material for: Health-seeking behaviour of older women: a protocol for a global scoping review
Source: BMJ Open. 2026 Nov 2;16(11):e127153. doi: 10.1136/bmjopen-2026-127153 (PMC13735260; doi:10.1136/bmjopen-2026-127153)
Supplement: Supplementary file 2 [file bmjopen-16-11-s001.docx]

**Health-seeking behaviour of older women: a protocol for a global scoping review**

**Supplementary file # 1**

**Complete search strategies for all databases, filters and limits:**

**Rationale for selecting these databases:**

Six databases were selected to capture evidence spanning health, ageing, psychology and the social sciences:

1. PubMed provides extensive coverage of biomedical, clinical and public health literature; Scopus offers broad multidisciplinary coverage and complements PubMed by indexing literature across health and social science disciplines
2. Scopus provides broad multidisciplinary coverage of peer-reviewed literature across health, social sciences and related fields, complementing the more specialized coverage of the other databases
3. PsycINFO was included to capture psychological, behavioural and psychosocial dimensions of healthcare-seeking
4. International Bibliography of the Social Sciences (IBSS) provides additional coverage of social science literature relevant to gender, ageing, socioeconomic circumstances and healthcare access
5. AgeLine was specifically selected because of its focus on ageing and older populations, increasing the likelihood of identifying relevant gerontological literature that may not be adequately captured through general health databases
6. Google Scholar complements the bibliographic databases by providing broader retrieval of interdisciplinary and potentially grey or less conventionally indexed literature.

Together, these sources were considered appropriate for capturing the individual, social, behavioural and health-system dimensions of older women's health-seeking behaviour that are central to the review.

**Search strategy:**

The search used database-specific free-text terms relating to older women and health-seeking behaviour; it combined synonyms within each concept using OR and connected the two concepts using AND. The table below presents the platform and its corresponding search strategy, the date for retrieving the titles and the yield per platfom:

| **Platform** | **Search strategy** | **Date retrieved on + no. of items** |
| --- | --- | --- |
| PubMed | ( "older woman"[tiab] OR "older women"[tiab] OR "elderly woman"[tiab] OR "elderly women"[tiab] OR "senior woman"[tiab] OR "senior women"[tiab] OR "aged woman"[tiab] OR "aged women"[tiab] OR "geriatric woman"[tiab] OR "geriatric women"[tiab] OR "aging woman"[tiab] OR "aging women"[tiab] OR "ageing woman"[tiab] OR "ageing women"[tiab] ) AND ( "health seeking"[tiab] OR "health-seeking"[tiab] OR "health seeking behaviour"[tiab] OR "health-seeking behaviour"[tiab] OR "health seeking behavior"[tiab] OR "health-seeking behavior"[tiab] OR "healthcare seeking"[tiab] OR "health care seeking"[tiab] OR "care seeking"[tiab] OR "care-seeking"[tiab] OR "help seeking"[tiab] OR "help-seeking"[tiab] OR "treatment seeking"[tiab] OR "medical care seeking"[tiab] OR "decision making"[tiab] OR "decision-making"[tiab] OR access*[tiab] OR accessibility[tiab] OR availability[tiab] OR availab*[tiab] OR affordability[tiab] OR affordab*[tiab] OR acceptability[tiab] OR accommodation[tiab] OR barrier*[tiab] OR facilitat*[tiab] OR "unmet need*"[tiab] OR utilization[tiab] OR utilisation[tiab] OR "service use"[tiab] ) | 15 June 2026  # 1890 |
| Scopus | ( "older woman" OR "older women" OR "elderly woman" OR "elderly women" OR "senior woman" OR "senior women" OR "aged woman" OR "aged women" OR "geriatric woman" OR "geriatric women" OR "aging woman" OR "aging women" OR "ageing woman" OR "ageing women" ) AND ( "health seeking" OR "health-seeking" OR "health seeking behaviour" OR "health-seeking behaviour" OR "health seeking behavior" OR "health-seeking behavior" OR "healthcare seeking" OR "health care seeking" OR "care seeking" OR "care-seeking" OR "help seeking" OR "help-seeking" OR "treatment seeking" OR "medical care seeking" OR "decision making" OR "decision-making" OR access* OR accessibility OR availability OR availab* OR affordability OR affordab* OR acceptability OR accommodation OR barrier* OR facilitat* OR "unmet need*" OR utilization OR utilisation OR "service use" ) ) AND PUBYEAR > 2020 AND ( LIMIT-TO ( LANGUAGE , "English" ) | 15 June 2026  # 2457 |
| PsycINFO | "( TI( "older woman" OR "older women" OR "elderly woman" OR "elderly women" OR "senior woman" OR "senior women" OR "aged woman" OR "aged women" OR "geriatric woman" OR "geriatric women" OR "aging woman" OR "aging women" OR "ageing woman" OR "ageing women" ) OR AB( "older woman" OR "older women" OR "elderly woman" OR "elderly women" OR "senior woman" OR "senior women" OR "aged woman" OR "aged women" OR "geriatric woman" OR "geriatric women" OR "aging woman" OR "aging women" OR "ageing woman" OR "ageing women" ) OR KW( "older woman" OR "older women" OR "elderly woman" OR "elderly women" OR "senior woman" OR "senior women" OR "aged woman" OR "aged women" OR "geriatric woman" OR "geriatric women" OR "aging woman" OR "aging women" OR "ageing woman" OR "ageing women" ) ) AND ( TI( "health seeking" OR "health-seeking" OR "health seeking behaviour" OR "health-seeking behaviour" OR "health seeking behavior" OR "health-seeking behavior" OR "healthcare seeking" OR "health care seeking" OR "care seeking" OR "care-seeking" OR "help seeking" OR "help-seeking" OR "treatment seeking" OR "medical care seeking" OR "decision making" OR "decision-making" OR access* OR accessibility OR availability OR availab* OR affordability OR affordab* OR acceptability OR accommodation OR barrier* OR facilitat* OR "unmet need*" OR utilization OR utilisation OR "service use" ) OR AB( "health seeking" OR "health-seeking" OR "health seeking behaviour" OR "health-seeking behaviour" OR "health seeking behavior" OR "health-seeking behavior" OR "healthcare seeking" OR "health care seeking" OR "care seeking" OR "care-seeking" OR "help seeking" OR "help-seeking" OR "treatment seeking" OR "medical care seeking" OR "decision making" OR "decision-making" OR access* OR accessibility OR availability OR availab* OR affordability OR affordab* OR acceptability OR accommodation OR barrier* OR facilitat* OR "unmet need*" OR utilization OR utilisation OR "service use" ) OR KW( "health seeking" OR "health-seeking" OR "health seeking behaviour" OR "health-seeking behaviour" OR "health seeking behavior" OR "health-seeking behavior" OR "healthcare seeking" OR "health care seeking" OR "care seeking" OR "care-seeking" OR "help seeking" OR "help-seeking" OR "treatment seeking" OR "medical care seeking" OR "decision making" OR "decision-making" OR access* OR accessibility OR availability OR availab* OR affordability OR affordab* OR acceptability OR accommodation OR barrier* OR facilitat* OR "unmet need*" OR utilization OR utilisation OR "service use" ) ) | 15 June 2026  # 353 |
| IBSS | (TITLE ("older woman" OR "older women" OR "elderly woman" OR "elderly women" OR "senior woman" OR "senior women" OR "aged woman" OR "aged women" OR "geriatric woman" OR "geriatric women" OR "aging woman" OR "aging women" OR "ageing woman" OR "ageing women") OR ABSTRACT("older woman" OR "older women" OR "elderly woman" OR "elderly women" OR "senior woman" OR "senior women" OR "aged woman" OR "aged women" OR "geriatric woman" OR "geriatric women" OR "aging woman" OR "aging women" OR "ageing woman" OR "ageing women") OR IF("older woman" OR "older women" OR "elderly woman" OR "elderly women" OR "senior woman" OR "senior women" OR "aged woman" OR "aged women" OR "geriatric woman" OR "geriatric women" OR "aging woman" OR "aging women" OR "ageing woman" OR "ageing women")) AND (TITLE("health seeking" OR "health-seeking" OR "health seeking behaviour" OR "health-seeking behaviour" OR "health seeking behavior" OR "health-seeking behavior" OR "healthcare seeking" OR "health care seeking" OR "care seeking" OR "care-seeking" OR "help seeking" OR "help-seeking" OR "treatment seeking" OR "medical care seeking" OR "decision making" OR "decision-making" OR access* OR accessibility OR availability OR availab* OR affordability OR affordab* OR acceptability OR accommodation OR barrier* OR facilitat* OR "unmet need*" OR utilization OR utilisation OR "service use") OR ABSTRACT("health seeking" OR "health-seeking" OR "health seeking behaviour" OR "health-seeking behaviour" OR "health seeking behavior" OR "health-seeking behavior" OR "healthcare seeking" OR "health care seeking" OR "care seeking" OR "care-seeking" OR "help seeking" OR "help-seeking" OR "treatment seeking" OR "medical care seeking" OR "decision making" OR "decision-making" OR access* OR accessibility OR availability OR availab* OR affordability OR affordab* OR acceptability OR accommodation OR barrier* OR facilitat* OR "unmet need*" OR utilization OR utilisation OR "service use") OR IF("health seeking" OR "health-seeking" OR "health seeking behaviour" OR "health-seeking behaviour" OR "health seeking behavior" OR "health-seeking behavior" OR "healthcare seeking" OR "health care seeking" OR "care seeking" OR "care-seeking" OR "help seeking" OR "help-seeking" OR "treatment seeking" OR "medical care seeking" OR "decision making" OR "decision-making" OR access* OR accessibility OR availability OR availab* OR affordability OR affordab* OR acceptability OR accommodation OR barrier* OR facilitat* OR "unmet need*" OR utilization OR utilisation OR "service use")) | 15 June 2026  # 115 |
| AgeLine | (TITLE("older woman" OR "older women" OR "elderly woman" OR "elderly women" OR "senior woman" OR "senior women" OR "aged woman" OR "aged women" OR "geriatric woman" OR "geriatric women" OR "aging woman" OR "aging women" OR "ageing woman" OR "ageing women") OR ABSTRACT("older woman" OR "older women" OR "elderly woman" OR "elderly women" OR "senior woman" OR "senior women" OR "aged woman" OR "aged women" OR "geriatric woman" OR "geriatric women" OR "aging woman" OR "aging women" OR "ageing woman" OR "ageing women") OR IF("older woman" OR "older women" OR "elderly woman" OR "elderly women" OR "senior woman" OR "senior women" OR "aged woman" OR "aged women" OR "geriatric woman" OR "geriatric women" OR "aging woman" OR "aging women" OR "ageing woman" OR "ageing women")) AND (TITLE("health seeking" OR "health-seeking" OR "health seeking behaviour" OR "health-seeking behaviour" OR "health seeking behavior" OR "health-seeking behavior" OR "healthcare seeking" OR "health care seeking" OR "care seeking" OR "care-seeking" OR "help seeking" OR "help-seeking" OR "treatment seeking" OR "medical care seeking" OR "decision making" OR "decision-making" OR access* OR accessibility OR availability OR availab* OR affordability OR affordab* OR acceptability OR accommodation OR barrier* OR facilitat* OR "unmet need*" OR utilization OR utilisation OR "service use") OR ABSTRACT("health seeking" OR "health-seeking" OR "health seeking behaviour" OR "health-seeking behaviour" OR "health seeking behavior" OR "health-seeking behavior" OR "healthcare seeking" OR "health care seeking" OR "care seeking" OR "care-seeking" OR "help seeking" OR "help-seeking" OR "treatment seeking" OR "medical care seeking" OR "decision making" OR "decision-making" OR access* OR accessibility OR availability OR availab* OR affordability OR affordab* OR acceptability OR accommodation OR barrier* OR facilitat* OR "unmet need*" OR utilization OR utilisation OR "service use") OR IF("health seeking" OR "health-seeking" OR "health seeking behaviour" OR "health-seeking behaviour" OR "health seeking behavior" OR "health-seeking behavior" OR "healthcare seeking" OR "health care seeking" OR "care seeking" OR "care-seeking" OR "help seeking" OR "help-seeking" OR "treatment seeking" OR "medical care seeking" OR "decision making" OR "decision-making" OR access* OR accessibility OR availability OR availab* OR affordability OR affordab* OR acceptability OR accommodation OR barrier* OR facilitat* OR "unmet need*" OR utilization OR utilisation OR "service use")) | 15 June 2026  # 85 |
| Google Scholar | With Google Scholar, a general keyword search was done and retrieved using Harzing’s Publish or Perish Software [<https://harzing.com/resources/publish-or-perish>].  Search terms used were "elderly women" ("health seeking" OR "care seeking" OR "help seeking" OR "treatment seeking" OR barriers OR "unmet needs" OR utilization). | 23 June 2026  # 500 |

**Filters:**

Five years
